# Molecular Mechanism and Approach in Progression of Meningioma

**DOI:** 10.3389/fonc.2020.538845

**Published:** 2020-09-11

**Authors:** Zhiwei Shao, Lihong Liu, Yanghao Zheng, Sheng Tu, Yuanbo Pan, Sheng Yan, Qichun Wei, Anwen Shao, Jianmin Zhang

**Affiliations:** ^1^Department of Hepatobiliary and Pancreatic Surgery, Second Affiliated Hospital, School of Medicine, Zhejiang University, Hangzhou, China; ^2^Department of Radiation Oncology, The Second Affiliated Hospital, Zhejiang University School of Medicine, Hangzhou, China; ^3^State Key Laboratory for Diagnosis and Treatment of Infectious Diseases, Collaborative Innovation Center for Diagnosis and Treatment of Infectious Diseases, The First Affiliated Hospital, College of Medicine, Zhejiang University, Hangzhou, China; ^4^Department of Neurosurgery, Second Affiliated Hospital, School of Medicine, Zhejiang University, Hangzhou, China; ^5^Brain Research Institute, Zhejiang University, Hangzhou, China; ^6^Collaborative Innovation Center for Brain Science, Zhejiang University, Hangzhou, China

**Keywords:** meningioma, mechanism, genetics, chromosomal abnormality, apoptosis, invasiveness, angiogenesis

## Abstract

Meningioma is the most common tumor of the central nervous system, most of which is benign. Even after complete resection, a high rate of recurrence of meningioma is observed. From in-depth study of its pathogenesis, it has been found that a number of chromosomal variations and abnormal molecular signals are closely related to the occurrence and development of malignancy in meningioma, which may provide the theoretical basis and potential direction for accurate and targeted treatment. We have reviewed advances in chromosomal variations and molecular mechanisms involved in the progression of meningioma, and have highlighted the association with malignant biological behavior including cell proliferation, angiogenesis, increased invasiveness, and inhibition of apoptosis. In addition, the chemotherapy of meningioma is summarized and discussed.

## Introduction

The meninges consist of the dura mater, arachnoid and pia mater, which envelope the surface of the brain and spinal cord. A tumor produced in the meninges is known as a meningioma (1). According to the latest statistical report of the Central Brain Tumor Registry of the United States (CBTRUS), meningioma has become the tumor with the highest rate of incidence of the central nervous system, accounting for approximately 37.1% (2). Meningioma is common in elderly patients, the median age of diagnosis being 66 years of age. Females are approximately 2.2-fold more likely to develop meningioma than males, except in atypical and anaplastic meningioma, where males outnumber female patients (2). The World Health Organization (WHO) classifies meningioma into grade I benign meningioma (>80%), grade II atypical meningioma (15–20%), and grade III anaplastic meningioma (1.0–3.0%) (2, 3). Meningioma can be further divided into 15 subtypes according to histopathology (4). Among them, meningothelial, fibroblastic, and transitional meningioma are the most common subtypes of the WHO (5). By comparing the immunophenotypes of normal arachnoid and meningiomas, research data indicate that arachnoid cap cells are likely to be the precursor cells of meningioma (6). Finally, a study established that prostaglandin D2 synthase (PGDS) positive arachnoid cells are the origin cells of meningiomas (7). Surgical resection can cure 70–80% of meningioma. However, atypical and anaplastic meningioma often have high recurrence rates, strong invasiveness and poor prognosis. Even after complete resection, the rate of recurrence of atypical and anaplastic meningioma is still as high as 50% and 80%, respectively (8). Furthermore, approximately 20% of meningioma with benign histology are likely to in fact be invasive and recurrent, which affects the treatment of the tumor (9). Previous studies have identified multiple molecular targets and genetic alterations that contribute to its progression, including those related to cell proliferation, increased invasiveness, angiogenesis, and inhibition of apoptosis. These molecular targets may be targeted in future to improve the therapeutic effect. Here, we summarize the molecular mechanisms that drive the biological behavior and relative medical treatment of meningioma.

## Cytogenetics

**FIGURE 1:**
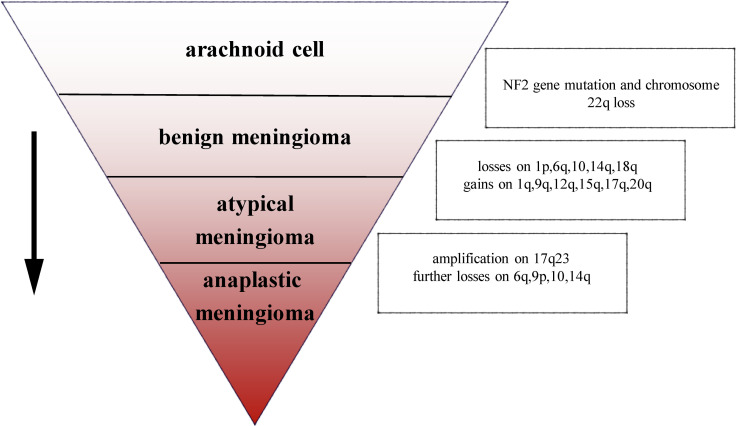
Chromosomal abnormalities in the progression of meningioma.

The most common chromosomal abnormality in meningioma is in chromosome 22, observed in 40–70% of grade I meningioma. Beyond the loss of chromosome 22, few other chromosomal abnormalities have been observed in benign meningioma (10). In an analysis of chromosome 22 in 44 sporadic meningiomas, researchers found that in 43 cases, all or part of the chromosome had been deleted, the majority of deletions occurring in the neurofibromatosis type 2 (NF2) region, suggesting that the mutation on NF2 leads to the occurrence of meningioma (11). In addition, a number of studies have demonstrated that loss of gene function of NF2 contributes to develop schwannoma, ependymomas, and malignant mesothelioma (12–14). Mice lacking NF2 are prone to develop cancers such as osteosarcoma, lymphoma, lung adenocarcinoma, hepatocellular carcinoma (HCC), and fibrosarcoma (15). Research has suggested that NF2 promotes contact inhibition and tumor suppression by suppressing mitogenic signaling at the cellular cortex (16). Therefore, the inactivation of NF2 plays an important role in early oncogenic events. Atypical and anaplastic meningioma exhibit a greater number of chromosomal abnormalities than benign meningioma, but the frequency of the NF2 gene mutations is almost the same as in benign meningioma, indicating that NF2 may not be related to the progression of meningioma (17). Loss of chromosome 1 is detected more often in atypical and anaplastic meningioma, and is the second most common deletion site in meningioma (1). The rate of deletion of 1p is significantly correlated with grade of meningioma (grade I meningioma: 13–26%; grade II: 40–76%; and grade III: 70–100%). Loss of 1p is also associated with malignant progression of meningioma (18). In atypical meningioma, chromosomal loss of 1p, 6q, 10, 14q, and 18q, and gains at 1q, 9q, 12q, 15q, 17q, and 20q have been observed. On the basis of the mutations described above, losses are more frequent at 6q, 9p, 10, and 14q with amplification of 17q23 in anaplastic meningioma (3) (Figure 1). In terms of chromosomal mutation and incidence of relapse in meningioma, researchers have used fluorescence *in situ* hybridization (iFISH) analysis on 302 meningioma samples, finding that alterations in 1p, 1q, 7, 9, 10, 14, 18, and 22 chromosome were significantly related to the incidence of relapse (19). Chromosomal variations provide a genetic basis for the stepwise progression of meningioma, which greatly assists in the diagnosis of grade and prognosis. Recent studies have also shown that the location of meningiomas is related to mutational profile. Compared with other anatomic locations, NF2 mutations are more common in the lateral regions and posterior skull base meningiomas, while the most majority of non-NF2 meningiomas often locate in the anterior, medial, or skull base regions (1, 20). For example, meningiomas with Smoothened (SMO) mutation are more likely to localize to the medial anterior skull base, near the midline (20). Meningiomas with Krueppel-like-factor 4 (KLF4)/TNF receptor-associated factor 7 (TRAF7) mutation often locate in the medial skull base and v-akt murine thymoma viral oncogene homolog 1 (AKT1)/TRAF7 mutation in the anterior skull base (21).

## Genetic Alterations With Clinical Prognosis

The genetic changes of meningioma is associated with the poor prognosis. A study have shown that activating mutations in the promoter of the telomerase reverse transcriptase (TERT) gene promote the aggressiveness of meningiomas and reduce the survival of patients (22). Patients with TERT promoter mutation had significantly shorter overall survival (53.8 vs 115.6 months; *P* = 0.0006). Another study also demonstrated that patients with TERT promoter mutation had a higher risk of recurrence and a shorter time to progression, regardless of WHO grade (23). In a study of 169 meningioma samples, Dystrophin-encoding and Muscular Dystrophy-associated gene (DMD) inactivation (by genomic deletion or loss of protein expression) was detected in 32% of patients with progressive meningiomas. Patients with DMD inactivation had significantly shorter overall survival than wild-type counterparts [5.1 years (95% CI 1.3–9.0) vs. median not reached (95% CI 2.9–not reached), *p* = 0.006] (24). Breast cancer (BRCA)1-associated protein-1 (BAP1) is a tumor suppressor gene encoding for a deubiquitylating enzyme. A study have demonstrated that BAP1-deficient meningiomas are more aggressive and have a poor prognosis (25).

## Molecular Mechanisms of Change in Biological Behavior

### Abnormal Cell Growth and Proliferation

**FIGURE 2:**
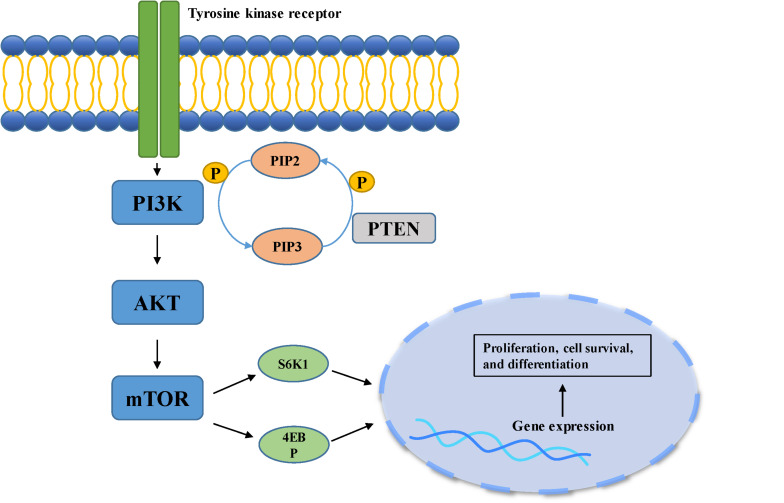
PI3K/Akt pathway is involved in cell growth, differentiation, and tumorigenesis. When the ligand binds to the membrane receptor, the receptor activates PI3K, which then catalyzes the formation of PI3P from PIP2 on the inner surface of the membrane. As the second messenger, PI3P further activated Akt. Akt can activate the downstream mTOR pathway, which can phosphorylate and activate S6K1 and 4EBP, and finally participate in gene expression.

The growth and proliferation of tumor cells are closely related to cell cycle dysregulation. For example, abnormal expression of cyclin, cyclin dependent kinases or their inhibitors often leads to enhanced proliferation and differentiation of meningioma cells (26, 27). The cyclin-dependent kinase inhibitor 2A (CDKN2A) gene can encode a variety of cell cycle regulating proteins including p16 [inhibitor of CDK4 (INK4a)] and p14 [alternative reading frame (ARF)] that inhibit the growth of tumor cells. Of these, the former principally inhibits the activity of cyclin dependent kinase 4 (CDK4) and 6 (CDK6), while the latter inhibits the degradation of tumor suppressor protein p53 that controls G1/S phase transition (8, 26). One study that compared the gene coding sequences of recurrent and non-recurrent meningioma found that changes in the CDKN2A gene were only observed in recurrent meningioma (28). The deletion of the CDKN2A gene promoted the malignant progression of meningioma, indicating poor outcome (29). Cyclin D1 (CCND1) controls the transition of the cell cycle, principally regulating the G1-S phase, and playing an important role in the transcription of tumor genes and cell proliferation (30). It has been reported that cyclin D1 is overexpressed in meningioma, and positively correlated with the degree of malignancy and rate of recurrence in meningioma. Knockdown of cyclin D1 expression in the meningioma cell lines IOMM-Lee and CHl57 demonstrated inhibition of the growth and proliferation of the cells (27). Forkhead box protein M1 (FoxM1) is a pro-mitotic transcription factor, which plays a positive regulatory role in the G1/S and G2/M transition of the cell cycle, ensuring smooth progression of mitosis (31). A separate study showed that an increase in FoxM1 expression can be observed in higher-grade meningioma, promoting the expression of β-catenin and cyclin D1, finally leading to proliferation and colony formation in meningioma cells (32). Similarly, overexpression of FoxM1 was also found in recurrent meningioma (32). A single transcriptome analysis, including the analysis of 280 human meningioma samples, demonstrated that FoxM1 plays a critical role in the proliferation of meningioma, indicating poor clinical prognosis (33). Cell cycle related proteins topoisomerase IIα and mitosin control the condensation and separation of mitotic chromosomes (34). A retrospective study of 160 meningioma patients found that patients with high topoisomerase IIα and mitosin expression suffered a higher risk of recurrence (35). Rapamycin [mammalian target of rapamycin (mTOR)] is mainly regulated by the phosphoinositide-3-kinase (PI3K)/protein kinase B (Akt) pathway, and is over-activated in both sporadic and hereditary brain tumors, related to cell growth, differentiation and tumorigenesis (36) (Figure 2). A study of human meningioma cell lines found that meningioma with over-expression of osteoglycin (OGN) exhibited higher cell proliferation, cell cycle activation and colony formation rate, activities closely associated with the PI3K/Akt/mTOR pathway (37). Inhibition of this pathway significantly suppressed abnormal cell proliferation and increased cell death. Yesilöz et al. analyzed 93 samples of skull base meningiomas, finding that over-activation of the mTOR signaling pathway was closely associated with the recurrence of meningioma (38). Another study demonstrated that the high expression of mTOR in atypical meningioma led to an increase in mitotic index, and meningioma which had high expression of mTOR exhibited worse prognosis (39).

### Increased Cell Invasiveness

**FIGURE 3:**
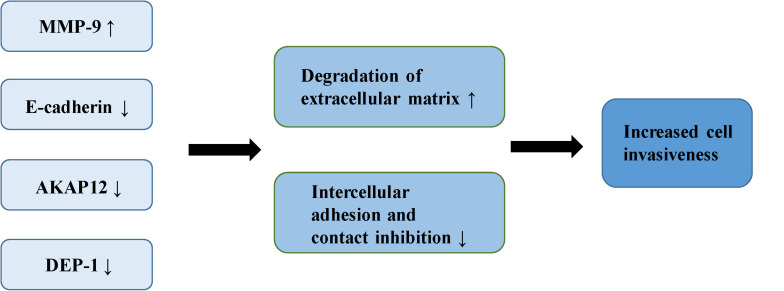
The mechanism of increased invasiveness in meningioma.

The adhesiveness of malignant cells is usually lower than that of normal cells. It was found that various cell adhesion molecules are expressed abnormally during the malignant invasion of meningioma. E-cadherin (encoded by CDH1 at 16q22.1) is a calcium-dependent adhesion molecule, mediating their interaction with epithelial cells (40). It relies on β-catenin to play a role in cell adhesion, which is considered an indirect regulator of the Wnt signaling pathway (10). E-cadherin and β-catenin comprise the E-cadherin/catenin complex that regulates cell adhesion and maintains cell polarity and stability (40). A low expression or absence of E-cadherin leads to the reduced formation of the complex, contributing to a decrease in adhesion between epithelial cells, the loss of intercellular connections, in addition to the weakening of contact inhibition, eventually leading to the uncontrolled growth of tumor cells and invasion of surrounding tissues (41, 42). Immunostaining of E-cadherin in 60 meningioma samples demonstrated that the expression of E-cadherin was down-regulated in 73% of meningioma. Moreover, E-cadherin has a more apparent down-regulatory effect in grade II and III meningiomas (43). Other studies of E-cadherin in meningioma have drawn similar conclusions. One study demonstrated negative expression of E-cadherin in all atypical meningioma, concluding that the increased invasiveness of meningioma is partly due to the loss of E-cadherin (44). Another study reported a positive correlation between the low expression of E-cadherin and the invasiveness of meningioma (45). Matrix metalloproteinases (MMPs) are zinc-dependent endopeptidases involved in the degradation of the extracellular matrix and tissue reconstruction, found to be closely associated with malignant invasion and distant metastasis of tumor cells (46). A number of reports have suggested that MMP-9 can promote the occurrence and development of tumors (47–50). Previous studies have shown that the expression of MMP-9 in meningioma is significantly correlated with the degree of malignancy and invasiveness. In the malignant meningioma cell lines IOMM-Lee and CH157-MN, MMP-9 has been shown to be significantly up-regulated with increased expression of the lncRNA LINC00460, leading to the progression of meningioma (51). The expression of MMP-9 in different grades of meningiomas has also been studied (52). Results of immunohistochemical analysis indicated that more MMP-9 was expressed in grade II and III meningiomas. Another study showed increased expression of MMP-9 in meningioma cells following irradiation (53). The authors confirmed increased invasiveness of the cells through spheroid migration and Matrigel invasion assays. The ability of cells to invade was reversed by the down-regulation of MMP-9. Petermann et al. found that loss of density-enhanced phosphatase-1 (DEP-1; a transmembrane protein-tyrosine phosphatase) reduced the adhesion of cell matrix, and enhanced the migration and invasive growth of meningioma cells (54). In the following experiments, Petermann et al. studied the relationship between DEP-1 and the invasiveness of meningioma cells (55). They found increased MMP-9 expression in DEP-1-depleted meningioma cells using zymography, suggesting that up-regulation of MMP-9 may contribute to the aggressive growth of meningioma. A recent study also demonstrated that the deficiency of DEP-1 promotes the progression of meningioma (56). A separate study demonstrated that the expression levels of tissue inhibitor of metalloproteinases-1 (TIMP-1) in invasive meningioma is significantly lower than that in non-invasive meningioma, possibly related to the inhibition of MMP-9 activity by TIMP-1 (57). The expression of A-kinase anchor protein 12 (AKAP12) increased in actively migrating cells, believed to play an important role in actin dynamics and actin filament-based migration (58). It is known that the expression of AKAP12 is inhibited in a number of human malignant tumors, such as melanoma, HCC, gastric cancer and BRCA (59–62). It has been found that knock-down of AKAP12 can promote the migration and invasion of meningioma cells, indicating that AKAP12 plays a possible role in inhibition of the progression of meningioma (63) (Figure 3).

### Angiogenesis

**FIGURE 4:**
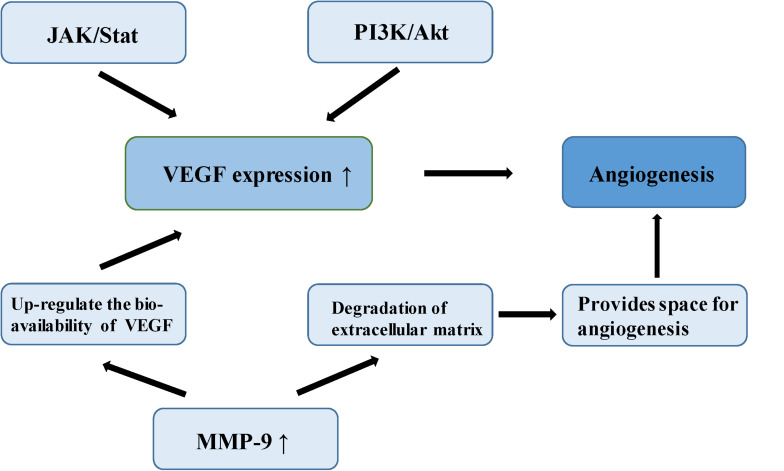
The mechanism of angiogenesis in meningioma.

Meningioma is a vascular-rich type of tumor, especially atypical and anaplastic meningioma, which are likely to relapse, suggesting that angiogenesis plays an important role in its malignant-type behavior (64). In a study of the molecular mechanism of tumor angiogenesis, Bergers proposed the theory of an “angiogenic switch,” that is, the dynamic balance of angiogenic and antiangiogenic factors which becomes broken, allowing the interaction of these factors to affect the biological behavior of meningioma, such as angiogenesis and invasion (65). Vascular endothelial growth factor (VEGF) is considered among the most important angiogenic factors, involved in multiple physiological and pathological pathways of angiogenesis. One study established that VEGF and its receptor are up-regulated in the hypoxic tissues of recurrent meningioma, inducing new angiogenesis to alleviate the hypoxia. Microvessel density (MVD) is closely related to poor prognosis in meningioma (66). In a study of 40 meningioma samples, a group of researchers found positive correlation between VEGF expression and pathological grade of meningiomas by immunohistochemistry (67). Hypoxia inducible factor-1 alpha (HIF-1α), an upstream regulator of a variety of signaling pathways, regulates many tumor metabolic processes, such as glycolysis, apoptosis, angiogenesis, and promotion of tumor growth (68). HIF-1α activates VEGF transcription via the PI3K/Akt signaling pathway, up-regulating VEGF expression and promotes endothelial cell proliferation in meningioma (69). In addition, over-expression of HIF-1α can increase the stability of VEGF and its receptor, and promote neovascularization (70). One study found that silencing of HIF-3α (an HIF-1α inhibitor) suppresses angiogenesis and proliferation in meningioma (71). As the upstream regulatory gene of VEGF, signal transducer and activator of transcription factor 3 (Stat-3), it regulates the expression of VEGF through the JAK/STAT signaling pathway combined with the promoter of VEGF, promoting tumor angiogenesis (72). Kwon et al. confirmed that MMP-9 is expressed to a significant level in meningioma, closely related to neovascularization and tumor cell migration (73). A study of malignant meningioma cell lines found that the expression of MMP-9 was silenced by small interfering RNA (siRNA) which could inhibit the formation of a capillary network (74). MMP-9 creates the space and stimulation for angiogenesis by promoting the degradation of extracellular matrix and releasing the VEGF embedded in the extracellular matrix. MMP-9 also enhances the binding function of VEGF and its receptor, and up-regulates the bioavailability of VEGF (74). In 1999, a number of researchers found a type of microcirculatory duct without endothelial cells in melanoma, termed tumor vasculogenic mimicry (VM). Blood is able to flow normally in these acellular tubular structures (75). The VM phenomenon has also been reported in malignant meningioma (76) (Figure 4).

### Inhibition of Apoptosis

**FIGURE 5:**
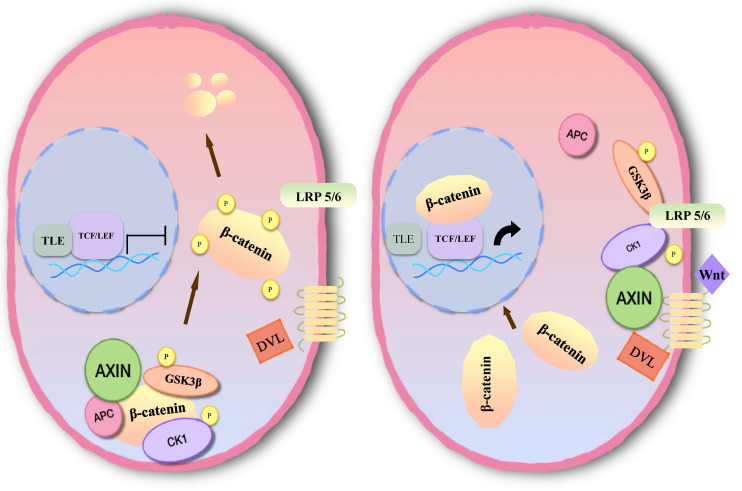
Molecular mechanism of Wnt/β-catenin signaling pathway activation.

Inhibition of apoptosis is closely associated with the occurrence, development and prognosis of tumors in meningioma. Several studies have found that the Wnt signaling pathway plays an important role in the development of meningioma, which is involved in apoptosis (77–79). The main pathway comprises three branches: the classical Wnt/β-catenin signal transduction pathway, the planar cell polarity (PCP) pathway and the Wnt-Ca^2+^ pathway (10). Members of the classical pathway include the extracellular Wnt factor, transmembrane receptor, β-catenin, glycogen synthase kinase-3β (GSK3β), adenomatous polyposis coli (APC), Axin, and casein kinase-1 (CK1) (10, 80). When the classical Wnt pathway is unactivated, GSK3β in the cytoplasm can phosphorylate β-catenin in the form of a complex with APC, Axin, and CK1 (81). By recognition of the phosphorylation site of β-catenin, β-TrCP can ubiquitinate β-catenin, then degrade it through a proteasome, and maintain a low level of β-catenin in cells (82). When Wnt signaling is activated, the Wnt protein binds with the extracellular domain of Frizzled (Fz). Through the synergistic effect of the low density lipoprotein receptor related protein (LRP 5/6), dishevelled protein (DVL) in the cytoplasm becomes translocated to the cellular membrane. DVL can phosphorylate GSK3β and lead to disintegration of the complex. In this case, β-catenin cannot be degraded, and a large number of free β-catenin aggregates in the cytoplasm and enters the nucleus (83). After β-catenin is combined with lymphoid enhancer factor (LEF)/T cell factor (TCF) to form a complex, suppression of LEF/TCF is reduced, and transcription of downstream target genes is specifically activated, including c-myc, cyclin D1, CD44, Bcl2, c-jun, etc. (84, 85) (Figure 5).

Multiple studies have shown that activation of the Wnt signaling pathway inhibits neural cell apoptosis (86–90). One study of meningioma found that lncRNA SNHG1 can inhibit apoptosis in BEN-1-1 and IOMM-Lee cells via the Wnt pathway, as found through the measurement of TUNEL-positive cells and caspase-3 activity (77). Knockdown of SNHG1 inhibited cell proliferation and promoted apoptosis. In another study, in which down-regulation of lncRNA LINC00702 inhibited Wnt signal activity in malignant meningioma, induction of apoptosis and decreased meningioma cell proliferation were observed (78). Unfortunately, there are few studies of the inhibition of apoptosis via the Wnt signaling pathway in meningioma. However, a large number of reports have been published concerning inhibition of apoptosis through the Wnt signaling pathway in glioma, neuroblastoma, spinal cord injury, and cerebral hemorrhage (85–91). Therefore, we can reasonably assume that the Wnt signaling pathway plays a role in inhibition of apoptosis in meningioma cells.

Many studies have explored the potential target of anti-apoptotic mechanisms in meningioma. One study assessed the expression of CD163 in 50 samples of meningioma (92). Researchers found that 48.5% and 71.4% of grade I and II meningioma, respectively, were positive for CD163. They also found human meningioma cell lines in which CD163 was overexpressed exhibited a decrease in apoptosis and hematopoietic cytokines, demonstrating that CD163 prevents apoptosis by production of granulocyte colony-stimulating factor (G-CSF). Other researchers compared the rate of apoptosis in meningioma between the FTS (a Ras inhibitor) group and control group by flow cytometric analysis, finding that apoptosis increased in the FTS group, indicating that Ras inhibition induced apoptosis (93). It has been reported that the expression of let-7d is down-regulated in meningioma, and that its overexpression induces apoptosis (94). In a recent study, researchers found a new oncogenic protein, N-myc downstream-regulated gene 4 (NDRG4), which is overexpressed in aggressive meningioma. After removal of NDRG4, the cells mostly died due to apoptosis (95).

## Medical Treatment for Meningioma

For the majority of patients with benign meningioma, surgical resection combined with stereotactic radiotherapy is effective in controlling the disease. However, no standardized treatment for recurrent or progressive meningioma has yet been published. Methods of treatment for meningioma include chemotherapy, molecular targeted therapy, immunotherapy, and hormone therapy, etc. (96).

### Molecular Targeted Therapy

A large number of studies have confirmed that the PI3K/Akt/mTOR pathway is overactive in meningioma, which has a close association with cell growth, differentiation and tumorigenesis, suggesting that mTOR is a potential therapeutic target for meningioma. In an *in vitro* experiment, eight mice with subcutaneous IOMM-Lee xenografts were treated with mTOR inhibitors (97). Growth of the tumor cells was significantly inhibited. A recent phase II trial of progressive meningioma achieved satisfactory results (98). Twenty patients with meningioma (including 18 non-benign meningioma) were treated with everolimus (an mTOR inhibitor) and octreotide (a somatostatin agonist). The overall PFS-6 was 55%, and a 6- and 12-month overall survival was 90% and 75%, respectively. Tumor growth rate decreased significantly after 3 months in 78% patients, and median tumor growth rate decreased from an initial 16.6% to 0.02%. Octreotide inhibits the phosphorylation of the PI3K/Akt pathway and activates tyrosine phosphatase, which can inhibit the proliferation of tumor cells *in vitro* (99). One study demonstrated that octreotide promotes the antitumor effect of everolimus in aggressive meningioma (100). Because a meningioma is a vascular-rich tumor, reduction in angiogenesis may be beneficial for treatment. VEGF is an important angiogenic regulatory molecule, which positively correlates with the pathological grade of meningioma. The expression levels of VEGF in atypical and anaplastic meningioma have been found to be 2 and 10 times higher than in benign meningioma, respectively (101). A retrospective study of 15 patients of atypical or anaplastic meningioma treated with bevacizumab (a VEGF inhibitor) found that the progression-free survival (PFS) rate was 43.8% at 6 months, with a median PFS of 26 weeks (102). Similarly, another retrospective study in which 14 patients with progressive meningioma were enrolled (including 5 patients with grade I meningioma) found that PFS at 6 months following treatment with bevacizumab was 86% (103). A phase II clinical trial evaluated the response of patients with advanced meningioma to combination treatment consisting of bevacizumab and everolimus (104). A total of 17 patients were treated, 15 of whom exhibited stable disease progression, with a median disease stabilization period of 10 months. An ongoing phase II clinical trial (NCT02847559) aims to test the efficacy of the combination of electric field therapy and bevacizumab in patients with recurrent or progressive meningioma. A separate phase II clinical trial recruited 36 patients with high-grade meningioma who were treated with sunitinib [a VEGF and platelet derived growth factor (PDGF) inhibitor], finding that median PFS was 1.4 and 6.4 months in VEGFR2 negative and positive patients, respectively (105). Despite the significant side effects (grade 3 or higher toxicity in 60% of patients), this clinical trial provides strong evidence for the potential targeting of VEGF in meningioma treatment.

### Other Treatments

Chemotherapy is not effective for the treatment of malignant meningioma, and is usually only used when the initial treatment fails or the tumor relapses (106). Timozolomide and irinotecan, which are efficient therapies for specific intracranial tumors, had no apparent therapeutic effect on meningioma (107, 108). Hydroxyurea is the most well-studied chemotherapeutic drug in advanced meningioma, and this exhibits a certain therapeutic effect (109). Interferon-alpha is a biologic agent able to inhibit DNA synthesis. In the early experiments of meningioma *in vitro*, interferon-alpha exhibited inhibitory activity toward the growth of tumor cells (110). A phase 2 study evaluated the efficacy of interferon-alpha in the treatment of WHO grade I recurrent meningioma, and found that the PFS rate was 54% at 6 months, demonstrating pharmaceutical activity (111).

Immunotherapy is a potential treatment regimen for malignant meningioma. It has been found that the expression of programmed death-ligand receptor (PD-L1) is increased in meningioma, especially in anaplastic meningioma (112). Therefore, two phase 2 clinical trials of treatments for high-grade meningioma are in progress, using nivolumab (NCT02648997), and pembrolizumab (NCT03016091), respectively. With the discovery of progesterone receptor (PR) in meningioma cells, the use of mifepristone (an antagonist of progesterone) in the treatment of meningioma patients has gradually attracted the attention of researchers (113). It has been reported that in 3 cases of meningioma treated with mifepristone, 2 cases exhibited radiological regression and 1 case was stable (114). A phase II clinical study showed moderate clinical improvement following treatment with mifepristone in meningioma (115). However, the first and only randomized phase III trial for unresectable meningioma demonstrated the opposite result. There was no significant difference in PFS in the mifepristone and placebo groups 10 months (95% CI 7–13 months) vs. 11 months (95% CI 6–18 months) (116). The failure of these chemotherapy agents in clinical studies is probably due to the wide molecular heterogeneity of meningiomas.

Unfortunately, at present, there are no strong clinical data showing that medical therapy has a significant effect on meningiomas. Moreover, the sample size of most relevant clinical trials is less than 20 patients. If we consider WHO grade, we will find that these clinical trials also include grade I meningiomas that do not normally require such second-line treatment. These data may influence the real beneficial effects of medical therapy on meningiomas. A greater number of randomized clinical trials are required to provide evidence for the medical treatment of meningioma.

## Conclusion

Meningioma is the most common tumor of the central nervous system, and most are considered benign. However, meningioma has a high recurrence rate, and the treatment of malignant meningioma is limited. Specially, the research on molecular mechanism and genetics treatment is insufficient compared with glioma. In this review, we focus on the molecular mechanism to elucidate the changes of biological behavior in the meningioma progression, including cell proliferation, increased invasiveness, angiogenesis, and inhibited apoptosis. Related signaling pathways and protein biomarkers may provide the direction and theoretical basis for the accurate targeted therapy of meningioma in the future.

## Author Contributions

ZS, YZ, ST, and YP drafted the manuscript. AS and JZ reviewed and modified the manuscript. LL, SY, and QW revised the manuscript. All authors agreed on the final version.

## Conflict of Interest

The authors declare that the research was conducted in the absence of any commercial or financial relationships that could be construed as a potential conflict of interest.
