# Corrigendum: Molecular Mechanism and Approach in Progression of Meningioma

**DOI:** 10.3389/fonc.2020.620376

**Published:** 2020-11-13

**Authors:** Zhiwei Shao, Lihong Liu, Yanghao Zheng, Sheng Tu, Yuanbo Pan, Sheng Yan, Qichun Wei, Anwen Shao, Jianmin Zhang

**Affiliations:** ^1^ Department of Hepatobiliary and Pancreatic Surgery, Second Affiliated Hospital, School of Medicine, Zhejiang University, Hangzhou, China; ^2^ Department of Radiation Oncology, The Second Affiliated Hospital, Zhejiang University School of Medicine, Hangzhou, China; ^3^ State Key Laboratory for Diagnosis and Treatment of Infectious Diseases, Collaborative Innovation Center for Diagnosis and Treatment of Infectious Diseases, The First Affiliated Hospital, College of Medicine, Zhejiang University, Hangzhou, China; ^4^ Department of Neurosurgery, Second Affiliated Hospital, School of Medicine, Zhejiang University, Hangzhou, China; ^5^ Brain Research Institute, Zhejiang University, Hangzhou, China; ^6^ Collaborative Innovation Center for Brain Science, Zhejiang University, Hangzhou, China

**Keywords:** meningioma, mechanism, genetics, chromosomal abnormality, apoptosis, invasiveness, angiogenesis

An author name was incorrectly spelled as Lilhong Liu. The correct spelling is Lihong Liu. 

The authors apologize for this error and state that this does not change the scientific conclusions of the article in any way. The original article has been updated.

